# The Value of the Preliminary Sciences

**Published:** 1908-12-26

**Authors:** 


### THE VALUE OF THE PRELIMINARY SCIENCES.

The medical curriculum of to-day has been criticised from many points of view, and often not with that impartial judicial clarity which it is necessary to bring to the discussion of matters on which there may be legitimate difference of opinion. There is a section that demands that the medical student should be emancipated from "the drudgery of learning a vast amount of useless stuff" and should be at liberty to devote himself to the practical part of his profession as soon as he has passed his Preliminary. There is, again, an opposite section that clamours for a greater insistence on subjects which have a bearing on medical science, though not usually reckoned as belonging essentially to it. The former class wishes an abrogation of the time devoted to the study of the preliminary sciences; the latter a prolongation of the whole period of study so as to give the student an opportunity of perfecting his knowledge in the preliminary subjects without trenching unduly upon the time at his disposal for practical and clinical work. To the student of medical history it is by no means a proved fact that the medical student of to-day has a harder task before him in preparing for his profession than his prototype had in the Dark Ages. Theology, at one time by no means a neglected part of preliminary study, astrology, and, alas! logic, we have thrown overboard, and it is no longer an axiom that the good physician must be an able dialectician. We have only changed the subjects; we have not materially altered or decreased the mass of work. The student has still to be diligent to satisfy his examiners within the prescribed time limits, and his studies are so confined that he is rarely satisfied with himself when he has gained the distinction of being a diplomate. The immediate value of the preliminary sciences is usually not recognised until clinical work, and actual bedside experience teach the student how much they are worth cultivating. The fault of our present system lies, perhaps, in this fact that during his first few terms the medical student is not taught the application of the anatomical, physiological, and other data that he learns. For that reason no one can glibly acquiesce in the proposed total separation of preliminary work from the medical curriculum as such. There is little chance nowadays for the student to verify anatomical facts in the post-mortem room, but there is always an opportunity, at least at hospitals to which medical schools are attached, for him to take his lesson first-hand in the dissecting room. Nor can it be argued that such verification is unnecessary, since the knowledge gained in studying the preliminary subjects should be ever present with the student. The true value of the preliminary sciences becomes manifest gradually. The application of this or that fact as occasion arises, through clinical work or otherwise, proves it fully to the student, and it is only after such demonstration of the practical utility of the initiatory sciences that he learns to estimate at its full worth the time he has spent in the laboratory or the museum. Properly to rank these preliminary sciences would mean a total reform in our medical curriculum as it is constituted at present. The demand for less drudgery may be met by more practical work and by a carefully thought-out scheme whereby the first year's student is enabled to see for himself the application of the facts he is actually studying. The desire for more systematic work at preliminary subjects by extending the whole period of study is less easy to satisfy, for the object of the curriculum is not the production of a finished scientist so much as the creation of a skilled class of professional men.

